# Perceptions of the Likelihood and Importance of Physical Activity Outcomes at 14 Years Affects Physical Fitness at 17 Years

**DOI:** 10.1111/cch.70276

**Published:** 2026-04-21

**Authors:** Amanda Timler, Paola Chivers, Helen Parker, Elizabeth Rose, Jocelyn Tan, Beth Hands, Mandy S. Plumb

**Affiliations:** ^1^ Paediatric Neurodevelopmental Disorder Group (PaNDer) University of New England Armidale NSW Australia; ^2^ Institute for Health Research University of Notre Dame Fremantle Australia; ^3^ School of Health Sciences University of Notre Dame Fremantle Australia; ^4^ Research Department Perth Children's Hospital, Child and Adolescent Health Service Nedlands Western Australia Australia; ^5^ School of Science and Technology University of New England Armidale NSW Australia; ^6^ Sports Performance, Exercise and Activity Research (SPEAR) University of New England Armidale NSW Australia; ^7^ University Department of Rural Health (UDRH) NT, Rural and Remote Health, College of Medicine and Public Health Flinders University Alice Springs Australia

## Abstract

**Objectives:**

The aims of this study were to examine whether the importance of physical activity and the likelihood of experiencing effects of physical activity changed between 14 and 17 years, and whether perceptions of importance and likelihood of physical activity in early adolescence align with aerobic fitness in late adolescence.

**Methods:**

Participants participated in the study at the age of 14 and then again at 17. They reported via survey at both time points on the likelihood and importance of being physically active over the next year, completing two 15‐item questionnaires. Cardiovascular endurance was measured in a laboratory setting on a bicycle ergometer using the PWC_170_ (Physical Working Capacity 170) at age 17 years.

**Results:**

A total of 1056 participants (554 girls and 502 boys) from the Raine Study Generation 2 (Gen2) took part. Their rating of the importance and likelihood towards physical activity was mainly influenced by intrinsic health (e.g., fit and healthy, losing weight, improving appearance) and social factors (e.g., having fun, spending time with friends) compared to extrinsic factors (e.g., change to win/compete, others making fun of them). Males positively rated more extrinsic factors compared to the females, with the rating of intrinsic factors such as ‘fit and healthy’ and ‘losing weight’ stable between 14 and 17 years. ‘Appearance’ was the only factor that increased in importance at 17 years compared to 14 years of age.

**Conclusion:**

Greater awareness of the factors that young adolescents consider as important and likely for their physical activity is important. These include having fun, spending time with friends and keeping fit and healthy. Sport coaches, physical education teachers and health promotion campaigns could consider targeted interventions focusing on these factors as an effective way of engaging youth.

## Summary

1. Early engagement in regular physical activity in early adolescence improves engagement in physical activity in late adolescence.
2. Parents, teachers and coaches should incorporate fun and enjoyable training strategies to encourage adolescents to remain physically active.
3. Physical activity training modules would benefit from including factors that motivate adolescents to remain physically active.

## 1 Introduction

High cardiorespiratory fitness when young has been associated with a healthier cardiovascular profile later in life (Myers et al. 2021; Nystoriak and Bhatnagar 2018) and therefore is an essential component for long term health and well‐being (Ji et al. 2024; Nystoriak and Bhatnagar 2018). Given lifelong physical activity levels and aerobic fitness are generally established in childhood and adolescence (Bardid et al. 2021), it is important to understand how to maximise motivations to be aerobically fit and active from an early age (Souza et al. 2021). In 2020, Guthold et al. (2020) identified that the majority of adolescents worldwide do not meet recommended physical activity levels, and unfortunately, these trends remain unabated (Duffey et al. 2021; van Sluijs et al. 2021).

Many factors impact exercise motivation and habits and subsequent physical fitness including genetic, social and environmental factors in adolescents (Ferreira Silva et al. 2022). These primary influences are important to understand an adolescent's ability to participate positively in play, games and sport, which may also influence their long‐term health and well‐being. Most authors examining the relationship between motivation and fitness report cross‐sectional studies with few linking adolescent motivations to actual fitness outcomes over time (Rosselli et al. 2020). Limited longitudinal studies have gathered data enabling an examination of factors during adolescence that may support or inhibit the development of aerobic fitness. Eime et al. (2016) considered changes in female adolescents' physical activity participation longitudinally and found a decrease in physical education and competitive sports participation, with an increase in non‐competitive physical activity such as walking, jogging or rollerblading during late adolescence. Therefore, despite years of research, insights into the development and maintenance of an individual's aerobic fitness including changes in their motivation towards being physically active remain elusive; this is particularly apparent among adolescents.

Adolescence is marked by wide variations in many cognitive and social factors, in particular sex differences and the timing and tempo of biological maturation. Motivations to become and remain involved in physical activity will vary but are compounded by an age‐related decline in physical activity linked with a widely observed drop‐out rate in sports participation (e.g., 33% drop‐out rate at 14 years) and the growing attraction of sedentary screen‐based activities (Vella et al. 2023). In general, females participate in less physical activity than males with a greater decline observed with age (Eime et al. 2020). Motivation has been identified as an important correlate of physical activity (Owen et al. 2014); therefore, understanding the motivational mechanisms that contribute to self‐determined physical activity levels and subsequent aerobic fitness is important for informing effective support.

Self‐determination theory argues that people can be intrinsically and extrinsically motivated towards physical activity and exercise (Deci and Ryan 2000). Ideally, a person is intrinsically motivated when they choose to be active (self‐determined behaviour) for the enjoyment and satisfaction of the activity itself. Examples include losing weight, having fun and spending time with friends. Intrinsic factors can lead to sustained health‐promoting behaviours if highly valued by the individual (Deci and Ryan 2000; Eime, Harvey, et al. 2013; Eime, Young, et al. 2013; Foley Davelaar 2021; Visek et al. 2015). On the other hand, extrinsically motivated physical activity is usually undertaken to obtain an external reward separate from the activity such as a trophy, money or fame (Vallerand and Losier 1999). Some of these extrinsic factors, if seen as controlling, will have a negative effect on a person's motivation to be active. Controlling factors that could negatively affect an individual's motivation to engage in physical activity could include the lack of time, some social influences, lack of energy or willpower, fear of being injured or lack of personal motivation (Vallerand and Losier 1999).

Motivation to remain physically active is often individualistic and may differ according to age and sex. For example, Hopkins et al. (2022) conducted a systematic review and found females continued to participate in sport if they found it enjoyable and felt confident. Therefore, understanding adolescent motivations and perceptions of the benefits of physical activity may be more effective in improving retention and engagement. Gender differences in motivation to participate in physical activity are important to consider as physical activity opportunities are likely to differ throughout adolescence. In a large study of 14‐year‐olds, Hands et al. (2015) found significant differences in the perceived likelihood and importance of physical activity as boys were more motivated by competition and winning (extrinsic motivations), whereas girls were more motivated by social interactions (intrinsic motivations). However, the focus of this paper was to understand the perceptions (likelihood and importance) of physical activity at 14 years, rather than change in motivation with age.

What is unknown is how perceptions or motivations about the benefits/importance and likelihood of outcomes to be achieved from being physically active and fit in early adolescence (14 years) are maintained with maturity and how these affect fitness outcomes during late (17 years) adolescence. Such information could inform school and community‐based intervention programs. The purpose of this study, therefore, is twofold. First, to examine whether perceptions about the importance of physical activity and the likelihood of experiencing effects of physical activity change between 14 and 17 years of age, and second, whether early adolescent perceptions about the importance and likelihood of physical activity align with aerobic fitness in late adolescence. For both purposes, the data are reported separately and compared between males and females to better understand motivations contributing to physical activity and fitness in late adolescence.

## 2 Methods

### 2.1 Participants

Participants of the Raine Study Gen2 (including 14‐year follow‐up and 17‐year follow‐up), which has been described in detail elsewhere (http://www.rainestudy.org.au). From 1989 to 1992, 2900 pregnant women reporting to antenatal clinics in Perth, Western Australia, were recruited into the study. The Raine Study Gen2 are broadly representative of the general Western Australian population with only the following differences 10.7% of parents never married (versus 9.8% in the general population), a lower proportion of fathers employed in managerial positions and a higher proportion employed in professional positions, 7.5% children were born < 37 weeks (versus 6.5%) and slightly more children born < 2500 g (versus 6.5%; Li et al. 2017; Straker et al. 2017). This paper reports data for the cohort at 17 years of age (554 girls and 502 boys), who completed the physical activity questionnaire at both 14 and 17 years and the aerobic fitness assessment at the 17‐year survey. Numbers for some variables differed as not all participants completed all assessment components.

## 3 Measures

### 3.1 Perceived Likelihood and Importance of Physical Activity Outcomes

At 14 years of age participants reported on the likelihood and importance of being physically active over the next year by responding to two 15‐item questionnaires from the New South Wales (NSW) Schools Fitness and Physical Activity Survey (Booth et al. 1997). The questionnaires were time efficient to administer and had strong psychometric properties of reliability and validity with this age group (Booth et al. 1997).

Questions asked about the perceived likelihood of outcomes of future participation in physical activity and the perceived importance of experiencing these effects. Questions included ‘make or keep me fit*’*, ‘keep me healthy’, ‘help me lose or control my weight’, ‘improve my appearance’, ‘keep my parents happy’ and ‘give me a chance to win something’. Responses were scored on a 7‐point Likert response format ranging from 0 (Extremely Unlikely/Least Important) to 6 (Extremely Likely/Most Important) for both positive and negative statements. For the analyses examining fitness outcomes at 17 years, the responses were converted to a 2‐point scale (0–3 = Least Important/Unlikely, neither; 4–6 = Most Important/Likely). For ease of interpretation and reporting of responses, the questions were notionally allocated to *Intrinsic social*, *Intrinsic health* and *Extrinsic* groups. *Intrinsic health* questions covered personal aspects about an individual's fitness, weight or injury. *Intrinsic social* questions included spending time with friends, studying better and having fun. Finally, questions allocated to the *Extrinsic* category included those where the individual receives a personal reward (wins, makes parents happy) or a negative experience (others make fun of them).

### 3.2 Aerobic Fitness

Cardiovascular endurance was measured in a laboratory setting on a bicycle ergometer using the PWC_170_ (Physical Working Capacity 170). This test provides an estimate of VO_2max_ and is considered a valid predictor of aerobic fitness in this age group (Rowlands et al. 1999). The heart rate (HR) at the three different loads was used to extrapolate the load required to lead to a HR of 170, using a linear regression designated the PWC_170_. Scores below 50 and above 250 were not included in the study following previous recommendations (Rowlands et al. 1999).

### 3.3 Procedures

For the 14‐ and 17‐year surveys, the questionnaires were mailed and completed by participants prior to attending for further assessments. At the 17‐year survey, the fitness assessments were carried out by the core group of the Raine Study Gen2 research staff in the fitness laboratory at the Institute for Child Research. They were trained health professionals and regularly checked for interrater reliability on all measures. Ethics approval was provided by the Human Research Committee at x.

### 3.4 Sample Size Estimate

The data for this project will be based on those with complete data sets for fitness measures, motor competence and physical activity at 17 years of age. Attrition due to long term follow‐up may affect the total sample size. Hands et al. (2015) examined motor competence between 10, 14 and 17 years of age and identified a complete sample size of 1215.

The study is planned to use a continuous response variable (Physical Work Capacity 170) from matched pairs of study subjects (those at 14 years followed up at 17 years). Prior data indicate that the difference in the response of matched pairs is normally distributed with standard deviation 30. If the true difference in the mean response of matched pairs is 3, we will need to study 787 pairs of subjects to be able to reject the null hypothesis that this response difference is zero with probability (power) 0.8.

## 4 Data Analysis

SPSS version 27 (SPSS Inc., Chicago, Illinois, United States) was used to undertake all analyses. Questionnaire responses and PWC_170_ scores were described using mean (M) and standard deviation (SD), with data normally distributed according to the Shapiro–Wilks test. The importance and perceived likelihood questionnaires were measured on a 7‐point Likert scale (0 being Last Important or Extremely Unlikely to 6 being Most Important or Extremely Likely). The 14‐year‐old and 17‐year‐old responses to the perceived likelihood of benefitting from being physically active in the future and the importance of being physically active with physical aerobic fitness at 17 years were first examined using paired sample *t*‐tests for the whole sample, males and females.

Independent samples *t*‐tests were conducted between the binary questionnaire categories for Importance (Least Important and Most Important) and Likelihood (Unlikely and Likely) and PWC_170_ results. Given sample sizes varied for some questions, Levene's test of inequality of error variances was completed. A significant result indicated that the variance between the two groups was not equal, and the more stringent *t*‐value was reported. Analyses were undertaken separately for males and females. Results were considered significant for *p* values less than 0.05. All assumptions for the independent samples *t*‐tests were met. The same data set was used for multiple statistical analyses and the Type I error probability associated with this test of this null hypothesis is 0.05.

## 5 Results

### 5.1 Importance and Likelihood Rankings at 14 and 17 Years

Male and female responses to the questionnaires at 14 years and 17 years are reported separately for the Importance and Likelihood statements, respectively in Tables 1 and 2. Responses were ranked from highest (most) to lowest (least) Importance or Likelihood at both 14 and 17 years. Where significant differences were observed, the direction of change is reported.

**TABLE 1:** The Importance of experiencing physical activity effects at 14 years and 17 years for a) males and b) females using a 7‐point Likert scale. Ranked in order of most to least importance at 14 years.

(a) Males							
The importance of experiencing effect of physical activity	*n*	14 years M (SD)	17 years M (SD)	*t*‐test, *p*	Direction change	Rank at 14 years	Rank at 17 years
Let me have a lot of fun	**492**	**5.23 (0.91)**	**5.08 (0.96)**	0.**004**	**↓**	1	1
Make or keep me fit	493	5.03 (0.94)	4.94 (1.08)	0.098		2	4
Keep me healthy	497	5.01 (0.95)	5.00 (1.03)	0.750		3	2
Help me spend time with friends	496	5.00 (0.90)	4.97 (1.04)	0.585		4	3
Feel good about self	494	4.98 (1.07)	4.94 (1.08)	0.529		5	5
Make my parents/carers happy	**494**	**4.75 (1.09)**	**4.32 (1.35)**	**< 0.001**	**↓**	6	7
Help me study and learn better	**495**	**4.74 (1.19)**	**4.53 (1.27)**	0.**001**	**↓**	7	6
Help me make new friends	497	4.33 (1.07)	4.28 (1.16)	0.471		8	9
Give me a chance to compete	**495**	**4.16 (1.29)**	**3.86 (1.58)**	**< 0.001**	**↓**	9	12
Help me lose/control weight	**497**	**4.10 (1.54)**	**3.77(1.71)**	**< 0.001**	**↓**	10	13
Prevent me doing things I like more	497	4.06 (1.26)	4.06 (1.35)	1.00		11	10
Give me a chance to win something	497	4.01 (1.36)	3.88 (1.49)	0.063		12	11
Improve my appearance	**496**	**4.00 (1.16)**	**4.29 (1.22)**	**< 0.001**	**↑**	13	8
Make a current injury worse	**495**	**3.70 (1.82)**	**3.48 (1.68)**	0.**034**	**↓**	14	14
If I tried others would make fun of me	**495**	**3.23 (1.59)**	**1.86 (1.69)**	**< 0.001**	**↓**	15	15

(b) Females							
The importance of experiencing effect of physical activity	*n*	14 years M (SD)	17 years M (SD)	*t*‐test, *p*	Direction change	Rank at 14 years	Rank at 17 years
Let me have a lot of fun	**550**	**5.30 (0.88)**	**5.15 (1.02)**	0.**001**	**↓**	1	1
Help me spend time with friends	**553**	**5.14 (0.90)**	**5.02 (1.13)**	0.**032**	**↓**	2	3
Feel good about self	**549**	**5.09 (0.99)**	**4.96 (0.99)**	0.**015**	**↓**	3	4
Keep me healthy	552	5.05 (0.92)	5.05 (0.94)	1.00		4	2
Make or keep me fit	549	4.97 (0.94)	4.95 (0.99)	0.658		5	5
Help me study and learn better	551	4.75 (1.09)	4.81 (1.14)	0.284		6	6
Make my parents/carers happy	**550**	**4.65 (1.13)**	**4.31 (1.41)**	**< 0.001**	**↓**	7	10
Help me make new friends	550	4.40 (1.00)	4.39 (1.13)	0.843		8	9
Help me lose/control weight	**552**	**4.32 (1.35)**	**4.58 (1.42)**	**< 0.001**	**↑**	9	8
Improve my appearance	**553**	**4.23 (1.16)**	**4.65 (1.15)**	**< 0.001**	**↑**	10	7
Prevent me doing things I like more	552	4.22 (1.18)	4.20 (1.33)	0.773		11	11
Give me a chance to compete	**553**	**3.56 (1.33)**	**2.97 (1.72)**	**< 0.001**	**↓**	12	14
Make a current injury worse	**553**	**3.54 (1.80)**	**3.25 (1.69)**	0.**002**	**↓**	13	12
If I tried others would make fun of me	**552**	**3.39 (1.46)**	**2.19 (1.77)**	**< 0.001**	**↓**	14	15
Give me a chance to win something	**552**	**3.38 (1.33)**	**3.01 (1.68)**	**< 0.001**	**↓**	15	13

*Note:* Light grey: intrinsic–social; white: intrinsic–health; dark grey: extrinsic.

**TABLE 2:** The perceived likelihood of outcomes for future participation at 14 years and 17 years of age for (a) males and (b) females. Ranked in order of most to least likely at 14 years.

(a) Males							
The perceived likelihood of outcomes for future participation in physical activity	*n*	14 years M (SD)	17 years M (SD)	*t*‐test, *p*	Direction change	Rank at 14 years	Rank at 17 years
Make or keep me fit	500	5.35 (0.84)	5.35 (0.84)	0.433		1	1
Keep me healthy	502	**4.95(1.02)**	**5.32 (0.94)**	**< 0.001**	**↓**	2	2
Feel good about self	501	4.93 (1.08)	4.88 (1.13)	0.214		3	3
Let me have a lot of fun	502	**4.87 (1.10)**	**4.72 (1.25)**	0.**006**	**↓**	4	4
Give me a chance to compete	499	**4.84 (1.19)**	**4.53 (1.44)**	**< 0.001**	**↓**	5	6
Give me a chance to win something	502	**4.80 (1.20)**	**4.43 (1.48)**	**< 0.001**	**↓**	6	7
Help me lose/control weight	501	**4.63 (1.33)**	**4.25 (1.60)**	**< 0.001**	**↓**	7	10
Make my parents/carers happy	500	**4.62 (1.14)**	**4.32 (1.26)**	**< 0.001**	**↓**	8	9
Help me make new friends	502	4.41 (1.29)	4.39 (1.32)	0.403		9	8
Improve my appearance	502	**4.30 (1.29)**	**4.70 (1.16)**	**< 0.001**	**↑**	10	5
Help me spend time with friends	502	**4.07 (1.35)**	**4.20 (1.40)**	0.**040**	**↑**	11	11
Help me study and learn better	500	**3.66 (1.34)**	**3.97 (1.51)**	**< 0.001**	**↑**	12	12
Prevent me doing things I like more	499	2.44 (1.65)	2.48 (1.67)	0.351		13	13
Make a current injury worse	501	**2.09 (1.81)**	**2.33 (1.68)**	0.**010**	**↑**	14	14
If I tired others would make fun of me	501	**0.96 (1.32)**	**0.70 (1.28)**	**< 0.001**	**↓**	15	15

(b) Females							
The perceived likelihood of outcomes for future participation in physical activity	*n*	14 years M (SD)	17 years M (SD)	*t*‐test, *p*	Direction change	Rank at 14 years	Rank at 17 years
Make or keep me fit	554	5.32 (0.82)	5.37 (0.87)	0.285		1	1
Keep me healthy	554	**4.95 (0.99)**	**5.35 (0.91)**	**< 0.001**	**↑**	2	2
Feel good about self	554	**4.89 (1.05)**	**5.06 (1.12)**	0.**002**	**↑**	3	3
Help me lose/control weight	554	**4.65 (1.27)**	**4.89 (1.29)**	**< 0.001**	**↑**	4	4
Let me have a lot of fun	553	**4.62 (1.19)**	**4.42 (1.31)**	**< 0.001**	**↓**	5	6
Give me a chance to compete	552	**4.53 (1.27)**	**3.86 (1.63)**	**< 0.001**	**↓**	6	10
Make my parents/carers happy	553	**4.43 (1.17)**	**4.12 (1.27)**	**< 0.001**	**↓**	7	9
Help me make new friends	552	**4.40 (1.30)**	**4.15 (1.38)**	**< 0.001**	**↓**	8	8
Give me a chance to win something	551	**4.40 (1.29)**	**3.64 (1.65)**	**< 0.001**	**↓**	9	12
Improve my appearance	554	**4.25 (1.27)**	**4.86 (1.18)**	**< 0.001**	**↑**	10	5
Help me spend time with friends	554	3.79 (1.41)	3.73 (1.45)	0.449		11	11
Help me study and learn better	551	**3.72 (1.26)**	**4.23 (1.37)**	**< 0.001**	**↑**	12	7
Prevent me doing things I like more	553	2.53 (1.52)	2.49 (1.55)	0.642		13	13
Make a current injury worse	553	1.99 (1.71)	2.14 (1.66)	0.095		14	14
If I tried others would make fun of me	554	0.82 (1.11)	0.73 (1.17)	0.151		15	15

*Note:* Light grey: intrinsic–social; white: intrinsic–health; dark grey: extrinsic.

For both males and females, the top five items for the Importance of effects were all intrinsic motivations (Table 1) and the top 5–6 ranked Importance of effects were also stable across 14 and 17 years (Table 1). Of those questions where responses changed significantly, most were less important at 17 years. The question that increased in Importance at 17 years for both males and females was ‘improve my appearance’. Females also ranked ‘help me control/lose weight’ as most Important. Of interest, both males and females ranked ‘let me have a lot of fun’ as most Important at both 14 and 17 years of age.

A similar pattern was evident for responses to the Likelihood of experiencing outcomes of future participation (Table 2). The top 5 questions were intrinsic factors and remained stable and similar between 14 and 17 years for both males and females. Keeping fit and healthy was ranked as most likely for both sexes. Both males and females considered it less likely that physical activity would let them have a lot of fun at 17 years, compared to 14 years, but it would improve their appearance.

### 5.2 Aerobic Fitness Levels at 17 Years and Importance Ratings at 14 Years

Participants were categorised into two groups according to their responses to the Importance (Most Important or least Important/Neither) of experiencing the effects of physical activity in the future. The aerobic fitness measures of each group at 17 years of age were then compared and reported separately for males and females (Tables 3 and 4). Unsurprisingly, at 17 years of age, the mean PWC_170_ measure for the males (M = 153.28, SD = 37.77) was significantly higher than for females (M = 100.27, SD = 24.10, *t* (900) = 27.11, *p* < 0.001).

**TABLE 3:** The comparison in importance of effects for future participation between males and females. Ranked in order of most to least likely at 14 years and PWC scores.

		*N*	Most Important M (SD)	*N*	Least Important/Neither M (SD)	*t*‐test, *p*
Keep me healthy	M	489	153.93 (37.87)	36	141.11 (32.70)	0.**024**
	F	491	100.51 (24.13)	24	95.22 (23.53)	0.147
Help me study and learn better	M	460	154.53 (38.40)	65	144.47 (32.86)	0.**013** *
	F	460	100.23 (23.74)	54	99.49 (26.16)	0.416
Improve my appearance	M	370	146.35 (39.77)	153	149.07 (37.47)	0.061
	F	416	100.06 (23.07)	99	101.13 (25.86)	0.347
Feel good about self	M	482	153.73 (38.24)	42	148.20 (31.33)	0.181
	F	481	100.51 (23.98)	31	97.08 (25.91)	0.221
Make or keep me fit	M	486	154.03 (37.63)	36	137.82 (36.41)	0.**006**
	F	485	100.53 (24.19)	28	95.49 (23.05)	0.142
Prevent me doing other things I like more	M	357	154.03 (37.63)	168	149.81(38.96)	0.077
	F	385	99.91 (24.37)	130	101.34 (23.34)	0.279
Help me lose/control weight	M	353	156.18 (38.09)	173	147.30 (36.75)	0.**006**
	F	401	100.93 (24.06)	113	98.02 (24.33)	0.129
If I tried others would make fun of me	M	239	155.69 (39.98)	284	151.15 (36.06)	0.086
	F	239	96.69 (20.86)	275	103.38 (26.29)	**< 0.001**
Make a current injury worse	M	310	159.06 (39.21)	213	144.67 (34.37)	**< 0.001**
	F	309	101.56 (24.67)	206	98.33 (23.16)	0.068
Let me have lots of fun	M	497	153.62 (38.21)	22	146.21 (23.98)	0.090*
	F	496	100.51 (24.20)	16	98.29 (19.28)	0.359
Make my parents/carers happy	M	453	153.09 (38.42)	70	153.62 (34.93)	0.458
	F	447	100.02(24.03)	67	102.45 (24.51)	0.220
Help me spend time with friends	M	496	154.13 (38.19)	28	137.03 (28.67)	0.**010**
	F	494	100.36 (24.24)	21	98.03 (21.20)	0.333
Help me make new friends	M	424	155.17 (38.47)	101	145.08 (34.28)	0.**008**
	F	435	100.26 (24.12)	80	100.33 (24.16)	0.489
Give me a chance to compete	M	387	157.79 (36.67)	136	140.44 (38.61)	**< 0.001**
	F	275	102.08 (24.63)	240	98.19 (23.36)	0.**034**
Give me a chance to win something	M	369	157.05 (37.86)	156	144.18 (36.46)	**< 0.001**
	F	245	101.25 (23.77)	269	99.39 (24.46)	0.193

* Levene's test was significant with two reported.

**TABLE 4:** The perceived likelihood of outcomes comparisons for future participation between males and females. Ranked in order of most to least likely at 14 years and PWC scores.

		*N*	Likely M (SD)	*N*	Unlikely/Neither M (SD)	*t*‐test, *p*
Keep me healthy	M	478	154.66 (37.42)	47	138.16 (38.22)	0.**002**
	F	479	100.92 (24.38)	34	90.10 (16.09)	**< 0.001** *
Help me study and learn better	M	288	154.14 (38.67)	235	151.79 (36.72)	0.240
	F	332	100.59 (24.46)	179	99.69 (23.36)	0.344
Improve my appearance	M	395	154.70 (35.82)	129	148.32 (42.98)	0.065*
	F	397	101.32 (24.70)	116	96.35 (21.39)	0.**025**
Feel good about self	M	470	153.61 (37.31)	54	148.89 (41.63)	0.192
	F	469	100.37 (24.48)	44	98.38 (19.24)	0.301
Make or keep me fit	M	509	153.61 (37.53)	15	136.68 (43.20)	0.**044**
	F	499	99.89 (23.98)	14	111.08 (25.13)	0.**043**
Prevent me doing other things I like more	M	151	151.49 (38.27)	371	153.85 (37.50)	0.258
	F	133	100.87 (25.63)	379	100.01 (23.53)	0.362
Help me lose/control weight	M	417	154.94 (37.61)	106	145.30 (37.08)	0.**009**
	F	434	100.99 (24.84)	79	95.85 (18.75)	0.**018** *
If I tried others would make fun of me	M	24	156.31 (35.02)	501	153.04 (38.00)	0.339
	F	16	110.20 (30.95)	497	100.06 (23.80)	0.**049**
Make a current injury worse	M	135	163.63 (39.28)	389	149.30 (36.43)	**< 0.001**
	F	127	104.07 (26.29)	386	99.16 (23.21)	0.**023**
Let me have lots of fun	M	462	154.54 (38.18)	63	143.92 (34.47)	0.**018**
	F	444	101.19 (24.39)	39	95.14 (21.40)	0.**026**
Make my parents/carers happy	M	426	154.48 (37.55)	100	148.06 (38.87)	0.064
	F	394	100.74 (24.66)	119	99.18 (22.12)	0.268
Help me spend time with friends	M	352	156.57 (38.41)	174	146.55 (35.86)	0.**002**
	F	311	100.53 (24.35)	202	100.15 (23.71)	0.430
Help me make new friends	M	415	156.57 (38.11)	111	140.87 (34.26)	**< 0.001**
	F	401	101.42 (23.96)	112	96.63 (24.26)	0.**031**
Give me a chance to compete	M	455	155.33 (38.02)	69	140.36 (34.51)	0.**001**
	F	434	100.58 (24.19)	78	99.33 (23.74)	0.337
Give me a chance to win something	M	457	155.18 (28.11)	69	140.74 (33.73)	0.**002**
	F	417	101.09 (24.59)	94	97.59 (21.60)	0.102

* Levene's test significant with three reported.

For the females, those who had felt that ‘others would make fun of them’ if they participated were significantly less fit at 17 years than those who rated that item as least important/neither. On the other hand, those males who valued physical activity as a way to ‘keep them fit and healthy’, ‘lose or control weight’, ‘provide an opportunity to compete or win something’, ‘help them study’ and ‘spend time with friends or make friends’ were significantly fitter than those who considered the benefits as less important or ambiguous. Females who valued the opportunity to compete were fitter than those who did not. Interestingly, the importance of ‘making a current injury worse’ was significant for males but not females. The males who considered this aspect important were fitter than those who did not.

### 5.3 Aerobic Fitness Levels at 17 Years and Likelihood Scores at 14 Years

Participants were categorised into two groups according to their responses to the Likelihood (Likely or Unlikely/Neither) of experiencing outcomes from future participation in physical activity. At 14 years, males who considered it likely that physical activity would ‘keep them healthy’, ‘keep them fit’, ‘help control weight’, ‘have a lot of fun’, ‘make their parents happy’, ‘spend time with friends and make new ones’ and ‘provide an opportunity to compete and to win’ something were all significantly fitter than those who considered these benefits Unlikely or were ambivalent (Table 3). For females, those who considered the Likelihood of physical activity ‘keeping them healthy and fit’, ‘having fun’ and ‘losing or controlling weight’ in the future were significantly fitter than those who did not. Similar to the Importance items, those who felt ‘others made fun of them’ if they tried to be active were significantly less fit. However, we have to acknowledge the smaller group comparisons between ‘Likely’ (24 males and 16 females) compared to ‘Unlikely/Neither’ (501 males and 497 females; Table 3). The concerned Likelihood about ‘making a current injury worse’ was similar for both sexes, with more in the fitter group expressing a greater Likelihood of this occurring.

## 6 Discussion

### 6.1 Summary of findings

Overall, the key finding of this study was intrinsic health (e.g., fit and healthy, losing weight, improving appearance) and social factors (e.g., having fun, spending time with friends) played a more significant role in shaping adolescents' motivation in physical activity compared to extrinsic factors (e.g., change to win/compete, others making fun), ultimately impacted by their level of fitness. Males rated a higher number of extrinsic factors as important compared to the females. The health and social intrinsic factors remained stable between 14 and 17 years, except for ‘appearance’ which increased in importance at 17 years compared to 14 years for both males and females.

Both males and females in this study indicated that intrinsic factors such as having fun, social reasons, and maintaining fitness and health were important for their engagement in physical activity, at both ages. In particular, the core factors of enjoyment and personal health were fundamental drivers of physical activity engagement throughout adolescence. Our results reinforce motivation as a significant predictor for physical activity enjoyment, including engagement in physical education and leisure activities, fitness levels, and social interactions among adolescents (Frömel et al. 2022; Jakobsen and Evjen 2018).

Interestingly, ‘improve my appearance’ increased in importance at 17 years for both males and females compared to 14 years. This finding is not surprising as during late adolescence, appearance becomes more important, with greater emphasis placed on peer comparisons and an increased concern with body image and group acceptance (Hopkins et al. 2022; Martins et al. 2021). This shift in perception often reflects changes in physical development and maturation (pre‐adolescence to adolescence), as older adolescents start to form a stronger sense of their own personal identity (Deci and Ryan 2000), and an increase in self‐awareness regarding their physical appearance (Harter 2012). Frömel et al. (2022) conducted a longitudinal study on motivation for physical activity among adolescents aged 15–19 years which showed a decline in later years for enjoyment, fitness and social motives. Males showed greater motivation for physical activity which was linked to enjoyment, competence, fitness and social motives compared to the females who valued appearance motives as important for their physical activity participation (Frömel et al. 2022).

The most Important intrinsic motivator, which remained stable across 14 and 17 years, was ‘to have fun’. This is encouraging, as it shows that enjoyment is a key driver of physical activity engagement from early to late adolescence. A number of studies around the world as well as across different sports and activities have also reported motivators for adolescents being physically active such as a sense of belonging to a team, personal enjoyment and learning, feeling healthy and physically competent, importance of having a friend and/or family member as part of the club, or for the ‘love of the game’ (Frömel et al. 2022; Jakobsson and Lundvall 2021; Perkinen et al. 2022). Adolescents who were motivated to be physically active did this to improve their physical and mental health, enhance physical fitness and develop social interactions, with higher levels of competence leading to greater enjoyment in physical activity participation (Frömel et al. 2022; Martins et al. 2021). Higher levels of aerobic fitness as observed in this study also lead to greater motivation to participate in physical activities. Overall, the key factors predicting physical activity involvement among adolescents include having positive attitudes and enjoyment towards physical activity, supportive parents and personally rating/valuing one's health (Hopkins et al. 2022; Rullestad et al. 2021).

Similar to the Importance rankings, the Likelihood to engage in physical activity remained stable across both ages. Intrinsic factors such as keeping fit and healthy increased these adolescents' engagement in physical activity. However, there was a decrease in the likelihood of ‘having fun’ between 14 and 17 years of age, which suggests that older adolescents are less likely to perceive physical activity as a recreational pursuit. Sport becomes more serious as training and level of commitment may lack flexibility and involvement becomes time consuming (Jakobsson and Lundvall 2021). Many studies have suggested that disengagement in physical activity is the result of higher levels of competition, greater focus placed on winning compared to having fun, experiencing less game time, greater involvement in more structured fitness routines, negative comments made by coaches, discomfort of their physical appearance, and pressures to perform more complex movement skills (Hopkins et al. 2022; Martins et al. 2021; Stodden et al. 2009). Consequently, these findings suggest that fitness organisations and sporting clubs should understand how motivators for physical activity involvement differ between younger to older adolescents. For many, it is preferable to maintain participation for enjoyment rather than for competition, by maintaining a sense of community rather than membership of a sporting team and participating in a fun, social manner.

The Likelihood for future participation in physical activity was primarily linked to keeping fit. Males and females who valued being fit ranked ‘making an injury worse’ as important and likely for their ongoing engagement in physical activity. This may be because they recognise the potential risks of developing/worsening an injury resulting in withdrawing from ‘the game’ (Frömel et al. 2022). This group may perceive greater benefits of being physically active (Martins et al. 2021), engaging in moderate to vigorous physical activity or specialised sporting programs, and meeting the recommended physical activity guidelines compared to those who are less active (Kudlacek et al. 2020). Therefore, increased consideration of injury management and maintenance while adolescents are participating in physical activities could help retain their engagement to remain fit and healthy. Adolescents who withdraw from participating in physical activity may perceive these activities as leading to an injury. This may be the result of lower levels of motor competence and preference to take part in other activities such as studying, working part time, spending time with friends and shopping or going out (Martins et al. 2021). Therefore, injury management and education about injury prevention may help this group participate in physical activity and sport for a longer duration if they have the right skills and information to manage this risk.

Higher aerobic fitness was observed in males, which is consistent with other studies (e.g., Rowlands et al. 1999). These findings also align with the physiological and maturation sex‐based changes that occur during puberty (Stodden et al. 2009). The higher aerobic outcomes commonly observed among male adolescents may be linked to the extrinsic factors (e.g., chance to complete or win, make parents happy) they rated as important compared to the females. Therefore, competition and winning continues to be important into late adolescence for males but not females, similar to Hands et al. (2015) who has also shown this relationship for 15‐year‐old males. Females are motivated more by fitness and unstructured physical activities compared to competitive sports (Eime et al. 2020). When considering the self‐determination theory (Deci and Ryan 2000), a person's motivation is affected by both the positive and negative consequences and nature of involvement in the activity (Vallerand and Losier 1999). This is linked to a sense of autonomy, competence and the influence of social events. Within a sporting context, motivational factors differ for males and females. Therefore, differences in aerobic fitness and motivation between sexes should be considered, as they are primary drivers for adolescent involvement in physical activity. More tailored approaches may be necessary to effectively promote physical activity and fitness among adolescents such as focusing on team and individual sports for males compared to improving parental support and encouraging involvement in unstructured physical activities such as dance or yoga for females (Kudlacek et al. 2020).

A positive relationship between an early adolescents' perceptions of their physical activity level was related to their aerobic fitness, but also differed between males and females. For example, males who were fitter, rated a larger number of intrinsic (spend time with friends and make new ones, losing weight, help with study) and extrinsic factors (make their parents happy, to compete and to win) as important for their fitness compared to their female counterparts who rated ‘fit and healthy’, ‘losing weight’ and ‘improving their appearance’ (intrinsic factors) as important for future physical activity engagement. Although not significant, ‘feeling good about oneself’ increased in Importance for females, but not males from 14 years to 17 years. Hopkins et al. (2022) highlighted that physical activity and sport participation positively influences the self‐concept of female adolescents, with athletic competence predicting physical activity and sports participation later in life. For the females in this study, the importance to engage in physical activity was linked to higher level of involvement in physical activities from a young age and weight control at 17 years. Many studies have highlighted that parental support and encouragement to participate in physical activity, finding specific activities they enjoy (e.g., yoga), developing a sense of belonging, finding an inclusive and safe environment to be physically active (Duffey et al. 2021; Martins et al. 2021) outweigh the perceived physical activity barriers for females. Elliott et al. (2019) facilitated focus groups with female adolescents participating in Australian Rules football and found their retention in this sport was influenced by finding an individual source of attraction such as ‘seeking the thrill’ or developing their identity, being in the optimal environment for kinship and adversity, and being a part of a successful team. Similarly, female adolescent basketball players continued to participate in this sport if they had developed a relationship with their team and had a strong sense of learning and improving the skill (Light and Pimenta 2020) compared to inhibiting factors such as negative attitudes and language by coaches, lack of a welcoming environment including bullying behaviours and lack of opportunity to play socially rather than just competitively (Sayers 2024).

Consequently, females in this study who were concerned about social stigmas (e.g., others made fun) had lower fitness levels. Some perceived physical activity barriers for female adolescents include a lack of time, lack of motivation (including psychological, emotional and cognitive factors) or energy, lack of access and lack of support from peers, parents and teachers (Duffey et al. 2021; Ferreira Silva et al. 2022; Hopkins et al. 2022; Rosselli et al. 2020; Zelenović et al. 2021), greater involvement in technology activities, concern regarding their safety, and body‐centred issues, as well as poor cultural norms, and limited portrayal of women athletes in the media (Frömel et al. 2022). In addition, feeling uncomfortable in front of others' especially males, being perceived as ‘uncool’ or not as a feminine activity (e.g., tomboy), and the discomfort from sportswear, sweating and current weight status influenced females' involvement in physical activity (Martins et al. 2021). The preference to participate in unstructured physical activity during late adolescence may directly relate to these barriers and their athletic competence (Eime et al. 2020; Hopkins et al. 2022) as female adolescents move away from organised, competitive settings to take part in non‐competitive and individual types of physical activity (Eime, Harvey, et al. 2013). These barriers highlight how negative social pressures (e.g., negative comments from coaches and parents) and academic study impact on sport and physical activity engagement for females (Hopkins et al. 2022). Therefore, positive re‐enforcement from coaches, teachers and parents for young female adolescents while participating in physical activities and feeling good about their involvement could improve physical activity engagement among this group.

Further studies need to consider clear sex‐based differences to better understand why female adolescents tend to be less physically active. Overall, there is a widely reported drop‐out rate in physical activity participation among Australian female adolescents (Eime et al. 2020; Duffey et al. 2021), with these rates increasing as adolescents become older (Vella et al. 2023). Opportunities to include alternative pathways, create positive environments (Elliott et al. 2022) and provide more specific sports and physical activities (not just mixed gendered sports) for all adolescents are some examples to improve physical activity engagement (Hopkins et al. 2022). The findings from this study emphasise the value intrinsic and some extrinsic factors have on adolescent motivation towards physical activity. In order to improve participation, sporting programs should include elements that incorporate a high level of enjoyment, social opportunities and focus on overall health. One way to achieve this could be for sporting organisations to review their accessibility for females, address any negative social interactions and clearly communicate the health and fitness benefits of physical activity engagement.

Greater focus should be placed on long‐term, positive physical fitness outcomes and motivators that are valued specifically for male and female adolescents (Kudlacek et al. 2020). The inclusion of health promotion initiatives such as incorporating the theory of planned behaviour (Norman et al. 2000) into social marketing campaigns could help change these perceptions, as personal attitudes (e.g., physical self‐perception), subjective norms (e.g., media representation of females in sport) influence perceived behavioural control (e.g., access and motivation to being physically active) towards physical activity. Therefore, future studies could focus on developing tailored interventions or specific physical activity programs in schools that focus on the motivators such as ‘fun’ and ‘fitness’ that drive adolescents to engage in physical activity (Martins et al. 2021). Specifically for females to be more confident in physical activity, programs should be created to decrease the negative social stigmas that prevent them from engaging in physical activity.

### 6.2 Strengths and Limitations

This longitudinal study is one of the first to consider how adolescents' perception towards the Importance and Likelihood of benefits may link to physical activity engagement. Some clear differences emerged between early and late adolescence and by gender. The large sample size (*N* = 1042) allowed for comparisons between age and gender to be considered, significant findings to be reported and met the expectations of the sample size calculation. The results from this study are also consistent with previous studies highlighting enjoyment, having fun and support from peers and family as key motivators for adolescents to be active. Only one other study (Hands et al. 2015) has considered physical activity motivation using the Study Gen2 cohort. This study adds to this body of work by looking across the developmentally critical phase of adolescence.

Some limitations must also be considered including the multiple statistical analyses on the same data set. In addition, some comparisons were made with smaller numbers between the groups which may limit the interpretation and generalizability of these findings; however, significant differences were seen, in particular for males displaying more extrinsic factors compared to the females.

## 7 Conclusion

Adolescents who engage in regular physical activity and consequently maintain a healthy level of aerobic fitness are most likely to be fit and active. Unfortunately, the study found that engagement in physical activity that is sufficient to build and maintain aerobic fitness reduced with age. Both intrinsic social and health factors were important for males and females in this study. It is vital, therefore, to consider the motivations for children and adolescents to participate in sport and physical activity and use this knowledge to empower teachers, coaches and parents to support adolescents becoming and staying fit. Based on this study, having fun, spending time with friends, keeping fit and healthy, losing weight, and improving appearance at 17 years (not 14 years) improved an adolescent's likelihood of engaging in physical activity. Males with higher aerobic fitness at 17 years had the highest perceptions towards the importance and likelihood of physical activity, compared to females and males with lower aerobic fitness. Adolescents with lower aerobic fitness at 17 years, regardless of gender, had a lower likelihood of being physically activity as they were considered others would make fun of them. Therefore, more effective strategies such as making physical activity fun and enjoyable could be put into place to promote sustainable physical activity engagement, leading to improved health and fitness outcomes.

## Author Contributions

**Amanda Timler:** writing – original draft, writing – review and editing. **Paola Chivers:** formal analysis. **Helen Parker:** conceptulaization, writing – original draft. **Elizabeth Rose:** conceptulaization, writing – original draft. **Jocelyn Tan:** writing – review and editing. **Beth Hands:** conceptulaization, writing – original draft. **Mandy S. Plumb:** writing – original draft, writing – review and editing.

## Funding

The Raine Study Gen2‐14 year follow‐up received funding from NHMRC (Sly et al., ID 211912), NHMRC Program Grant (Stanley et al., ID 003209) and The Raine Medical Research Foundation. The Raine Study Gen2‐17 year follow‐up was funded through a NHMRC Program Grant (Stanley et al., ID 353514).

## Ethics Statement

Ethics approval was provided by the Human Research Committee at the Princess Margaret Hospital (ref: 2019/RA/4/20/5722).

## Conflict of Interests

The authors declare no conflicts of interest.

## Data Availability

A de‐identified copy of the data is available upon request by the corresponding author.
